# Impact of CD14 on Reactive Oxygen Species Production from Human Leukocytes Primed by *Escherichia coli* Lipopolysaccharides

**DOI:** 10.1155/2019/6043245

**Published:** 2019-03-03

**Authors:** Dmitry S. Kabanov, Olga Yu. Vwedenskaya, Marina A. Fokina, Elena M. Morozova, Sergey V. Grachev, Isabella R. Prokhorenko

**Affiliations:** ^1^Department of Molecular Biomedicine, Federal Research Center “Pushchino Scientific Center for Biological Research of the Russian Academy of Sciences”, Pushchino 142290, Russia; ^2^Department of Pathology of the Institute of Pharmacy, SBAE I. M. Sechenov's First Moscow State Medical University of Russian Healthcare Ministry, Moscow 119811, Russia

## Abstract

Lipopolysaccharides (LPS) from Gram-negative bacteria prime human polymorphonuclear neutrophils (PMNs) via multicomponent receptor cluster including CD14 and MD-2·TLR4 for the enhanced release of reactive oxygen species (ROS) were triggered by bacterial derived peptide *N*-formyl-methionyl-leucyl-phenylalanine (fMLP). In this study, we investigated the impact of CD14 on LPS-induced priming of human PMNs for fMLP-triggered ROS generation (respiratory or oxidative) burst. Monoclonal antibodies against human CD14 (mAbs) as well as isotype-matched IgG2a did not influence significantly fMLP-triggered ROS production from LPS-unprimed PMNs. Anti-CD14 mAbs (clone UCHM-1) attenuated LPS-induced priming of PMNs as it had been mirrored by fMLP-triggered decrease of ROS production. Similar priming activity of S-LPS or Re-LPS from *Escherichia coli* for fMLP-triggered ROS release from PMNs was found. Obtained results suggest that glycosylphosphatidylinositol-anchored CD14 is the key player in LPS-induced PMN priming for fMLP-triggered ROS production. We believe that blockade of CD14 on the cell surface and clinical use of anti-CD14 mAbs or their Fab fragments may diminish the production of ROS and improve outcomes during cardiovascular diseases manifested by LPS-induced inflammation.

## 1. Introduction

Oxidative stress is a major contributing factor to the high mortality rates associated with several diseases and can sometimes potentially lead to lethal systemic disorders induced by LPS toxicity during bacteremia and septic shock. The excessive release of reactive oxygen species (ROS) from immune cells and/or inadequate antioxidant defense are the main reasons of oxidative stress development [1, 2]. ROS play a significant role in the pathogenesis of a myriad of inflammatory and cardiovascular diseases, such as diabetes, atherosclerosis, asthma, Alzheimer's disease, psoriasis, rheumatoid arthritis, and aging [3, 4]. Sepsis is associated with the excessive ROS production in both the circulation and the affected organs. In pathological complications, such as acute lung injury, excessive ROS production by neutrophils may influence vicinal cells of endothelium thereby contributing to the inflammatory tissue injury [5]. The release of “priming” agents such as proinflammatory cytokines TNF-*α* or IL-1*β* by immune cells significantly upregulates the production of superoxide anion radical (О_2_^·–^) during the immune response [1].

In sepsis, there are several potential ROS sources, including the mitochondrial respiratory electron transport chain, activation of xanthine oxidase as a result of ischemia-reperfusion, and the respiratory burst associated with immune cell activation. In fact, activated immune cells produce О_2_^·–^ as a cytotoxic agent as part of the respiratory burst via the action of membrane-bound NADPH oxidase on molecular oxygen. The assembly of NADPH oxidase is upregulated in PMNs exposed to bacterial LPS [6, 7]. So after LPS interaction with PMNs, they alter their resting state into the primed one and subsequent interaction of these primed PMNs with bacteria or their molecular patterns (PAMPs) immediately causes the substantial ROS release [7, 8]. There is a considerable body of evidence for redox imbalance and oxidative stress in sepsis, demonstrating increased markers of oxidative damage during this process [9–11]. In this case, septic shock can be understood as severe sepsis with cardiovascular failure.

The increased number of activated neutrophils producing ROS during sepsis may be destructive to the host tissue [6, 12]. Recruited to inflammatory sites or in conditions characterized by ischemia-reperfusion, PMNs produce ROS and cytokines. Proinflammatory cytokines are involved in cardiac muscle dysfunction and in the complex syndrome of heart failure [13, 14]. PMNs have been shown to infiltrate eroded or ruptured plaques obtained from patients with acute coronary syndromes and participate in the pathogenesis of lethal myocardial reperfusion [15–18]. Listed disorders in the cardiovascular system might be complicated by the primed state of PMNs and amplified ROS production during bacteremia and sepsis. Understanding how PMNs become primed should help to develop strategies to maintain the crucial balance between their beneficial and detrimental effects. Now, the great progress in identifying PMN proteins involved in signaling from cell surface receptors to the assembly of NADPH oxidase has been made [19].

The membrane-anchored form of CD14 (mCD14), Toll-like receptor 4 (TLR4), and TLR4-associated adaptor protein MD-2 are essential receptors involved in PMN priming by LPS [20–23]. The importance of myocardial TLR4 as the main player in cardiac dysfunction during the acute phase of LPS-driven septic shock in mice has been shown earlier [24]. It has been also proposed that the *β*_2_ integrin CD11b/CD18 plays the significant role in LPS signaling because it can influence TLR4-mediated cell activation [25]. Understanding of the molecular mechanisms by which the main receptors of LPS-induced receptor cluster, namely, mCD14, MD-2·TLR4, and CD11b/CD18, regulate PMN priming by LPS for amplified О_2_^·–^ release triggered by fMLP is an important scientific and medical task in the field of septic shock and oxidative stress development. We previously have shown that mAbs against human CD11b (clone ICRF 44, IgG1) or human TLR4 (HTA125, IgG2a) did not change fMLP-triggered ROS generation from LPS-primed PMNs [7, 8]. This is in agreement with the results of Qing et al., who showed that the anti-CD11b (LM2/1, IgG1) or anti-CD18 (60.3, IgG2a) mAbs did not inhibit LPS binding to PMNs [26]. As it has been mentioned above, surface glycoprotein mCD14 takes part in the assembly of LPS-induced receptor cluster. Therefore, the present study has been undertaken to investigate what is the contribution of mCD14 in LPS-induced priming of human PMNs for fMLP-triggered ROS release.

CD14 is a glycosylphosphatidylinositol- (GPI-) anchored cell surface receptor of PMNs. It presents LPS to and signals via MD-2·TLR4 and the MyD88-dependent signaling pathway [21, 27]. TLR4 independent of mCD14 cannot mobilize all of the adapter proteins that it requires for full signaling activity [22, 28]. CD14 is essential for LPS-induced activation of phospholipases and MAPKs [29]. It plays a role not only in TLR4 but also in Toll-like receptor 2 signaling [30, 31]. Nevertheless, the role of CD14 in LPS-induced PMN priming for fMLP-triggered ROS production is not well known. Therefore, using anti-CD14 mAbs (UCHM-1, IgG2a) consisting of the F(ab′)_2_ region specific to human CD14 and the intact mouse-reproduced Fc region, we investigated the impact of mCD14 in fMLP-triggered ROS production by LPS-primed PMNs.

## 2. Materials and Methods

### 2.1. Reagents

Luminol, *N*-formyl-methionyl-leucyl-phenylalanine, Percoll, Purpald reagent, mouse anti-human CD14 mAbs (clone UCHM-1), and S-LPS from *Escherichia coli* were purchased from Sigma-Aldrich (USA). Dextran 25GR was obtained from Fluka (Switzerland). The control isotype-matched mouse IgG2a (MCA929) was purchased from Serotec (UK). Re-LPS from *E. coli* JM103 were extracted according to [32]. Re-LPS were verified by testing for the presence of the oxidation products of 3-deoxy-D-*manno*-octulosonic acid (KDO) and L- or D-glycero-D-*manno*-heptoses using Purpald reagent [33]. The levels of contaminating proteins and nucleic acids in Re-LPS were monitored spectrophotometrically using Ultraspec 7000 (Biochrom, UK) [34]. The purity of extracted Re-LPS was also examined by SDS PAGE. Electrophoresis of Re-LPS *E. coli* followed by silver staining did not reveal any visible bands in the middle to upper regions of the gel, indicating the absence of contaminating proteins [35]. Thus, 98% pure Re-LPS *E. coli* were obtained. Before each test, S-LPS or Re-LPS in aqueous solution diluted to the desired working concentration was sonicated for 5 min. Phosphate-buffered saline (PBS) at pH 7.4 and solution for the determination of luminol-enhanced chemiluminescence (138 mM NaCl, 0.01 mM CaCl_2_, 5.9 mM KCl, 5 mM NaHCO_3_, 1 mM Na_2_HPO_4_, 1 mM MgSO_4_, 10 mM HEPES, 5.5 mM glucose, pH 7.4) were passed through a nitrocellulose filter with a pore size of 0.20 *μ*m.

### 2.2. PMN Isolation

Heparinized venous blood (19 ml) and 1 ml of whole blood without anticoagulant from seven consenting healthy volunteers were obtained under clinical conditions. Blood studies were approved by the local institutional medical ethnical committees in accordance with the standards laid down in the 1964 Declaration of Helsinki (adapted by World Medical Association General Assembly, Fortaleza, Brazil, 2013). PMNs were isolated from the whole blood in accordance with [36]. Briefly, heparinized (10 U/ml) whole blood was spun at 300 g for 15 min to separate cells from the plasma. A platelet-rich plasma layer was carefully aspirated and centrifuged at 2500 g for 15 min for the production of platelet-poor plasma (PPP). Then, 1.9 ml of 6% dextran solution in 12 ml of 0.9% NaCl was added to the cell pellet mixed gently and allowed to stand for 30 min for red blood cell sedimentation. After that, the upper phase enriched in leukocytes was centrifuged at 275 g for 6 min, and the resulting pellet was transferred into the PPP. This suspension of leukocytes in PPP was layered on a Percoll density gradient (1.077 g/ml) and fractioned by centrifugation for 15 min at 750 g. The pellet, containing PMNs and red blood cells, was resuspended in hypotonic erythrocyte-lysing solution (1 mM Na_2_-EDTA, 150 mM NH_4_Cl, 10 mM NaHCO_3_, pH 7.7) and then washed twice by PBS. The final cell preparation contained 96-98% PMNs. The cells were determined to be >97% viable by the exclusion of trypan blue.

The blood samples obtained without anticoagulant were allowed to clot at 37°C 60 min. Then, the clots were removed by spun at 200 g for 5 min at 4°C. 100–150 *μ*l resulting serum was used for autologous supplementation of the solution for luminol-enhanced chemiluminescence (CL) measurement.

### 2.3. Incubation of PMNs with Anti-CD14 Antibodies or IgG2a

To establish the role of CD14 in PMN priming by LPS, the isolated cells were preexposed to mAbs directed against human CD14 (or to IgG2a as isotype-matched control) for 30 min before LPS stimulation. Control (intact) cells did not contact these immunoglobulins. Subsequently, the cells were washed twice in PBS and resuspended in Ca^2+^-free solution for CL measurement. Then, the cells were kept under resting condition for 1 h at 4°C.

### 2.4. PMN Priming by LPS

When the resting stage was complete, control (intact) cells and cells preexposed to anti-CD14 mAbs or IgG2a were placed in chemiluminometer's chambers containing solution for luminol-enhanced CL that was supplemented with 0.01 mM CaCl_2_ and autologous serum (2%). We used serum as the source of LPS-binding protein (LBP). It has been shown that LBP regulates LPS-mediated events by forming complexes with LPS and delivering them to monocyte mCD14 [37, 38]. In the absence of serum, higher concentrations of LPS and longer incubation times are required to PMNs to potentiate fMLP-triggered ROS production [39]. A serum factor shifts the LPS dose-response curve to lower concentrations, and 30 min of incubation is well enough to achieve full priming by LPS [40]. When the experimental system was designed, the cells were allowed to be adapted in chambers for 5 min at 37°C. The priming state of PMNs was achieved by the addition of S-LPS or Re-LPS (100 ng/ml) followed by continuous gentle shaking for 30 min at 37°C [7].

### 2.5. Determination of ROS Production

The respiratory burst response of PMNs was measured using luminol-enhanced CL in the twelve-channeled CHEMILUM-12 elaborated at the Institute of Cell Biophysics (Pushchino, Russia) [41]. The measurements were done at 37°C with an acquisition frequency of 1/2.5 sec from 12 samples simultaneously. The PMNs in the chemiluminometer's chambers were continuously gently shaken with or without LPS under CL-monitored conditions for 30 min at 37°C. To activate the system, 1 *μ*M fMLP was added to the cells and the light emission was recorded continuously for 20 min. Total ROS production from control and LPS-primed PMNs within the first 50 sec after fMLP stimulation was expressed as chemiluminescence arbitrary units (AU) and calculated as the area under the curve of millivolts versus time (Integral of CL response, a.u.·sec). The values of CL response (integral) were calculated using software designed by A. A. Grinevich (Institute of Cell Biophysics, Russia).

### 2.6. Statistical Analysis

The data were analyzed using the statistical package STATISTICA 7.0. The statistical significance between appropriate groups was calculated using Wilcoxon's signed-rank nonparametric date analysis. Differences were considered to be significant when *p* < 0.05.

## 3. Results

### 3.1. fMLP-Triggered ROS Production from Human PMNs

**Figure 1:**
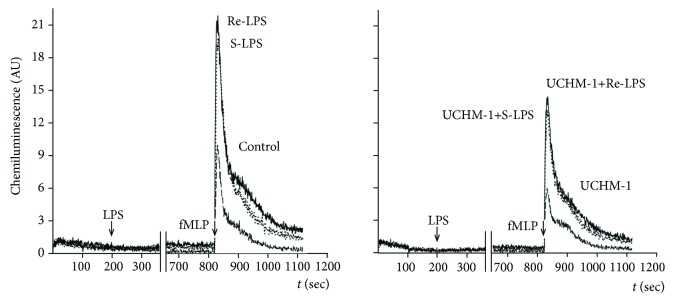
Influence of anti-CD14 mAbs (UCHM-1) on ROS production from LPS-primed fMLP stimulated human PMNs. Control (intact) PMNs and PMNs preexposed to anti-CD14 mAbs had been primed by S-LPS or Re-LPS *E. coli* (100 ng/ml) for 30 min at 37°C, and then, ROS generation was triggered by fMLP.

In all performed experiments, ROS generation from fMLP-stimulated PMNs was immediately observed (Tables 1 and 2). These results confirm that the viable cells were used in our study. The ROS production from isolated PMNs varied considerably from donor to donor both in the magnitude of respiratory burst and in the total amount of generated ROS (Tables 1 and 2), revealing differences in their functional states. The median value (M) and interquartile range of CL response (integral) observed in control (unprimed) PMNs stimulated by fMLP were 36.5 a.u.·sec and 34.2–75.5 a.u.·sec, respectively. The fast and relative slow phases of fMLP-triggered ROS production from unprimed PMNs are well distinguishable in Figure 1. The most dramatic changes in these two phases of ROS generation have been seen when PMNs were the first LPS primed and then stimulated by fMLP.

### 3.2. Influence of S-LPS or Re-LPS Glycoforms on fMLP-Triggered ROS Production from Human PMNs

The structure of S-LPS can be formally divided into the three regions: extensive polysaccharide region (O-antigen), which is connected with the hydrophobic lipid A region through core oligosaccharide. Gram-negative bacteria produce LPS of different glycoforms (S or R) depending on the genetically determined biosynthesis of O-antigen polysaccharide and core oligosaccharide regions. As the result, Re-LPS consists of the lipid A region covalently linked to several residues of KDO and devoid of the core region and O-antigen [42, 43]. In order to investigate the impact of O-antigen in LPS priming, we used S-LPS or Re-LPS isolated from *E. coli*. These LPS have almost identical lipid A structures but differ in the length of their carbohydrate parts.

In the time course of PMN priming by S-LPS or Re-LPS, we did not observe any CL unless fMLP was added (Figure 1). The values of fMLP-triggered CL in the samples of LPS-unprimed PMNs were chosen as the controls. The median values of CL response (integral) estimated in the samples of control and LPS-primed PMNs are summarized in Table 1. From the comparison of fMLP-triggered ROS production from S-LPS or Re-LPS-primed PMNs, it might be formally concluded that the Re-LPS had more potent priming potency in comparison with that of S-LPS (Figure 1). However, these differences did not reach a statistical significance (M 75.6 vs. 69.3 a.u.·sec; *p* = 0.3). Thus, under used experimental conditions, similar priming potency of S-LPS or Re-LPS for fMLP-triggered ROS production from human PMNs has been established. Unlike control unprimed but fMLP-triggered PMNs, the decay of luminol-enhanced CL of S-LPS- or Re-LPS-primed and fMLP-stimulated PMNs detected during the first ~300 sec did not reach the baseline (Figure 1). This phenomenon may be explained by the levels of intracellular calcium concentration [Ca^2+^]_i_. It has been shown that LPS-primed neutrophils have increased levels of resting [Ca^2+^]_i_ and retained them for a longer period of time in comparison with unprimed ones [44]. It is likely that, by maintaining elevated levels of [Ca^2+^]_i_ for a longer period of time after initial stimulation, LPS-primed cells may be more responsive to secondary stimulation by fMLP that was mirrored in our study by CL decay. Control PMNs, unexposed to LPS, showed no priming when the cells were stimulated with fMLP.

### 3.3. Influence of Isotype-Matched IgG2a on fMLP-Triggered ROS Production from Re-LPS-Unprimed and Re-LPS-Primed PMNs

To block the CD14 on the cell surface of PMNs, the full mouse mAbs against human CD14 have been used. Therefore, the effect of the Fc part of mouse-derived anti-CD14 mAbs on fMLP-triggered ROS production from LPS-unprimed as well as LPS-primed PMNs must be verified. Thus, PMNs were exposed to isotype-matched mouse IgG2a followed by fMLP stimulation. The comparison of fMLP-triggered ROS release from Re-LPS-unprimed but IgG2a exposed PMNs revealed a negligible suppressive effect of IgG2a on fMLP-triggered ROS production (Table 2). Taking this fact into consideration, the effect of IgG2a on fMLP-triggered ROS generation from Re-LPS-primed PMNs was examined. When PMNs have been the first exposed to IgG2a followed by Re-LPS priming, the fMLP-triggered ROS production was two-fold higher than those from control and IgG2a-exposed cells (Table 2). Keeping in mind that isotype-matched IgG2a causes a negligible suppressive effect on fMLP-triggered ROS release from LPS-unprimed cells (Table 2), we concluded that observed amplification of fMLP-triggered ROS production from PMNs that the first had been preexposed to IgG2a and then primed by Re-LPS completely attributed to the priming effect of Re-LPS.

### 3.4. Influence of Anti-CD14 mAbs on fMLP-Triggered ROS Production from LPS-Unprimed PMNs

We next investigated the contribution of anti-CD14 mAbs UCHM-1 to fMLP-triggered ROS production without LPS priming. The incubation of PMNs with anti-CD14 mAbs led to different ROS releases during cell response to fMLP (Table 1). In most cases, there were no significant differences between ROS production from PMNs unexposed or exposed (M 36.5 vs. 33.1 a.u.·sec; *p* = 0.6) to anti-CD14 mAbs and then stimulated by fMLP. Unlike PMNs primed by S-LPS or Re-LPS, the decay of CL reached baseline within ~300 sec of detection time only in the samples of unprimed PMNs exposed to anti-CD14 mAbs and then triggered by fMLP.

### 3.5. Influence of Anti-CD14 mAbs on fMLP-Triggered ROS Production from S-LPS- or Re-LPS-Primed PMNs

The data summarized in Table 1 and presented in Figure 1 clearly show that while the anti-CD14 mAbs have no effect on fMLP-triggered ROS production from unprimed PMNs, they significantly downregulated fMLP-triggered ROS production from S-LPS- or Re-LPS-primed PMNs by 18% or 13%, respectively (Figure 1). Unlike mAbs against CD14, the anti-TLR4 (HTA125) or anti-CD11b (ICRF 44) mAbs did not inhibit the priming action of S-LPS or Re-LPS from *E. coli* [7, 8]. Obtained results support the thesis that mCD14 is the key player in LPS-driven PMNs priming for fMLP-triggered ROS generation.

## 4. Discussion

The mechanisms for LPS-induced priming of PMNs are poorly understood. It has been proposed that the amplified ROS release from LPS-primed PMNs is the result of the cross-talk of at least two intracellular signaling pathways. The first LPS-driven MD-2·TLR4- and MyD88-dependent pathway recruits intracellular adaptor proteins such as TIRAP/MAL, IRAK, TRAF6, and TAK1; among them, kinase TAK1 is linked to MAPK signaling cascades [21]. MKK3-dependent phosphorylation of p38 MAPK was observed after 20 min of PMN exposure to LPS [45]. Note that CD14 could be associated with the nucleotide regulatory G_i*α*2_ subunit of G proteins [46]. The second fMLP-triggered signaling pathway is realized via G protein-coupled formyl peptide receptor 1 (FPR1) leading to the activation of PI3K*γ*, p38 MAPK, and ERK1/2 kinases [47–49]. Then, the activation of PI3K*γ* and/or phospholipase C*γ* induces Ca^2+^ mobilization and generation of diacylglycerol (DAG), which in turn activates protein kinase C [50]. Finally, the phosphorylation of essential p67^phox^ and p47^phox^ components of NADPH oxidase by activated p38 and ERK1/2 MAPKs leads to the assembly of NADPH oxidase and О_2_^·–^ generation [47]. So it has been concluded that translocation of G_i*α*2_ proteins and intracellular components of NADPH oxidase to PMN plasma membrane are the potential mechanisms underlying PMN priming by LPS [51, 52].

By using anti-CD14 mAbs and LPS amplified fMLP-triggered ROS production as a functional measure of cell priming by LPS, we established the system in which impact of CD14 in LPS-induced signaling via MD-2·TLR4 could be investigated. First, we examined the effect of anti-CD14 mAbs or isotype-matched IgG2a on fMLP-triggered ROS release from control LPS-unprimed PMNs. To block the CD14 receptor, we used full mAbs, so the involvement of Fc receptor gamma- (Fc*γ*Rs-) mediated events cannot be ruled out definitely. Participation of Fc*γ*Rs in PMN activation for ROS production has been shown in many studies [53, 54]. Among human Fc*γ*Rs, the Fc region of mouse IgG2a is recognized by CD64 and CD32 receptors with the highest and the moderate affinities, respectively [55]. We revealed that anti-CD14 mAbs (UCHM-1) as well as isotype-matched IgG2a did not influence significantly fMLP-triggered ROS production from unprimed PMNs (Tables 1 and 2). These data are in a good agreement with previous findings showing that anti-CD14 mAbs MY4 (IgG2b) did not influence fMLP-triggered О_2_^·–^ generation [40, 46]. The negligible suppressive effect of IgG2a on fMLP-triggered ROS generation from unprimed PMNs (Table 2) might be associated with the inhibitory CD32B isoform of the CD32 receptor [50]. However, UCHM-1 antibodies did not exhibit the same effect as isotype-matched IgG2a on fMLP-triggered ROS production from LPS-unprimed PMNs. These indicate some differences in mechanisms underlying the influence of UCHM-1 or IgG2a on the fMLP-triggered signaling pathway in human PMNs.

Then, we could show that anti-CD14 mAbs UCHM-1 downregulate fMLP-triggered ROS production from LPS-primed human PMNs (Figure 1). The obtained result is consistent with the data of Yasui et al. (1992) and Troelstra et al. (1997), who investigated the effect of another anti-CD14 mAbs MY4 (IgG2b) or 60bca (IgG1) on ROS generation from control and LPS-primed fMLP-stimulated PMNs [46, 56]. The inhibition of LPS-driven PMN priming by anti-CD14 mAbs UCHM-1 was specific, since neither anti-TLR4 (HTA125) nor anti-CD11b (ICRF 44) mAbs decreased the ROS production from LPS-primed human PMNs in our previous studies [7, 8]. So our findings support the thesis that mCD14 is the key player in LPS-driven PMN priming for fMLP-triggered ROS generation [26, 40].

It is necessary to note that certain anti-CD14 mAbs such as 63D3 or biG6 could not prevent LPS-induced signal transduction in human monocytes [46, 57–60] suggesting that UCHM-1 and MY4 may bind to different epitopes on CD14, with the UCHM-1 or MY4 (SAVEVEIHAGG) epitopes being crucial for LPS-induced signal transduction than those of 63D3 or biG6 Abs. Since anti-CD14 mAbs UCHM-1 only suppressed fMLP-triggered ROS production from LPS-primed PMNs but did not block LPS priming completely, we proposed the presence of PMN multiple LPS-binding sites; among them, some are affected and some are unaffected by these mAbs [61].

In spite of the fact that our study does not elucidate the mechanisms underlying the effects of anti-CD14 mAbs (UCHM-1) on LPS-induced priming of human neutrophils, several possible explanations are conceivable. Based on the above mentioned results and our own study, it is possible that the reduction in LPS priming may be associated with the UCHM-1-dependent steric interference between LPS and LPS-binding site(s) on CD14 [62] preventing the LPS-induced receptor cluster assembly. The second reason is the downregulation of surface TLR4, which is a consequence of TLR4 internalization mediated by anti-CD14 mAbs [63]. Note that anti-CD14 mAbs (MY4) have been found to be the most effective at the reduction of surface TLR4 as well as mCD14. In addition, anti-CD14-dependent shedding of mCD14 from the neutrophil surface as the mechanism attenuating their priming by LPS may be also proposed [64, 65].

Although LPS is the major ligand of CD14, recent data indicate that it can also interact with other ligands including Gram-positive bacteria such as lipoteichoic acid, soluble peptidoglycan, muramyldipeptide, polymannuronic acid, and lipoarabinomannan [30, 31] providing a rationale for blocking CD14 function to reduce the consequences of bacterial-induced inflammation [64, 65]. In accordance, treatment with anti-CD14 (IC14) during human endotoxemia strongly inhibited LPS-induced proinflammatory cytokine release, whereas the release of anti-inflammatory cytokines such as soluble TNF receptor type I and IL-1Ra was only delayed [64, 65]. In addition, IC14 treatment also inhibited LPS-induced IL-8, MCP-1, and MIP-1*β* chemokine production, while LPS-induced MIP-1*α* levels were neither inhibited nor delayed [65].

## 5. Conclusion

From the results presented here, several conclusions can be drawn regarding the regulation of ROS production by LPS-primed and fMLP-stimulated PMNs. First, S-LPS or Re-LPS revealed almost the same priming activity for fMLP-triggered ROS production from human PMNs. Second, UCHM-1 mAbs against human CD14 attenuated LPS-induced priming of human PMNs as it had been mirrored by fMLP-triggered ROS production. Third, isotype-matched IgG2a had a negligible suppressive effect on fMLP-triggered ROS generation from unprimed PMNs. Obtained results support the thesis that mCD14 is the key player in LPS-driven PMN priming for fMLP-triggered ROS production. We believe that blockade of CD14 on the cell surface and clinical use of anti-CD14 mAbs or their Fab fragments may diminish ROS production and improve outcomes during cardiovascular diseases manifested by LPS-induced inflammation.

## Figures and Tables

**Table 1:** The influence of anti-CD14 mAbs (UCHM-1) and S-LPS or Re-LPS on fMLP-triggered ROS production by human PMNs.

	Total ROS production during the first 50 sec (integral of CL response, a.u.·sec)					
	Control, fMLP (*n* = 7)	S-LPS, fMLP (*n* = 7)	UCHM-1, S-LPS, fMLP (*n* = 7)	Re-LPS, fMLP (*n* = 7)	UCHM-1, Re-LPS, fMLP (*n* = 7)	UCHM-1, fMLP (*n* = 7)
Median (range) and interquartile (range) values	36.5	75.6^∗^	62.2^∗∗^	69.3^∗^	60.2^∗∗^	46.3†
	34.2–75.5	60.5–183.9	58.9–65.7	63.0–175.3	52.5–78.4	35.2–69.3

^∗^ *p* < 0.05 statistically significant vs. control PMNs. ^∗∗^*p* < 0.05 statistically significant vs. LPS-primed but not mAbs-exposed PMNs. ^†^*p* > 0.05 statistically insignificant vs. control PMNs.

**Table 2:** The influence of isotype-matched mouse IgG2a and Re-LPS on fMLP-triggered ROS production by human PMNs.

	Total ROS production during the first 50 sec (integral of CL response, a.u.·sec)		
	Control, fMLP (*n* = 7)	IgG2a, fMLP (*n* = 7)	IgG2a, Re-LPS, fMLP (*n* = 7)
Median (range) and interquartile (range) values	63.7	60.2	129.2^∗^
	53.7–97.2	48.4–73.4	109.7–147.2

^∗^ *p* < 0.05 statistically significant vs. control PMNs.

## Data Availability

The data used to support the findings of this study had been included in the article body and available from the corresponding author upon request.

## Abbreviations

AU: Arbitrary units
IL-1Ra: Interleukin-1 receptor antagonist
IRAK: IL-1 receptor-associated serine/threonine protein kinase
MAPK: Mitogen-activated protein kinases
MKK3: Mitogen-activated protein kinase kinase 3
MCP-1: Monocyte chemoattractant protein 1
MD-2: Myeloid differentiation factor 2 lymphocyte antigen 96
MIP-1*α*/*β*: Macrophage inflammatory protein 1 *α*/*β*
MyD88: Myeloid differentiation primary response gene 88
NADPH oxidase: Nicotinamide adenine dinucleotide phosphate oxidase
PI3K*γ*: Phosphoinositide 3-kinase gamma
TAK1: Transforming growth factor *β*-activated kinase
TIRAP or MAL: Toll-interleukin 1 receptor (TIR) domain-containing adaptor protein or MyD88 adapter-like
TRAF6: Tumor necrosis factor receptor-associated factor 6.
